# Non-Hebbian plasticity at C-fiber synapses in rat spinal cord lamina I neurons

**DOI:** 10.1016/j.pain.2013.04.011

**Published:** 2013-08

**Authors:** Asami Naka, Doris Gruber-Schoffnegger, Jürgen Sandkühler

**Affiliations:** Department of Neurophysiology, Center for Brain Research, Medical University of Vienna, Spitalgasse 4, 1090 Vienna, Austria

**Keywords:** Pain, Synapse, Calcium, C-fiber, Intrinsic membrane properties, Long-term potentiation (LTP), Non-Hebbian

## Abstract

Current concepts of memory storage are largely based on Hebbian-type synaptic long-term potentiation induced by concurrent activity of pre- and postsynaptic neurons. Little is known about non-Hebbian synaptic plasticity, which, if present in nociceptive pathways, could resolve a number of unexplained findings. We performed whole-cell patch-clamp recordings in rat spinal cord slices and found that a rise in postsynaptic [Ca^2+^]_i_ due to postsynaptic depolarization was sufficient to induce synaptic long-term potentiation (LTP) in the absence of any presynaptic conditioning stimulation. LTP induction could be prevented by postsynaptic application of the Ca^2+^ chelator BAPTA (1,2-bis(o-aminophenoxy)ethane-N,N,N′,N′-tetraacetic acid), the L-type voltage-gated calcium channel (VGCC) antagonist nifedipine, and by postsynaptic application of the NMDA receptor antagonist MK801. This indicates that synaptic potentiation was induced postsynaptically by Ca^2+^ entry likely via L-type voltage-gated Ca^2+^ channels (VGCC) and via NMDA receptor channels. The paired pulse ratio and the coefficient of variation remained unchanged in neurons expressing LTP, suggesting that this form of synaptic potentiation was not only induced, but also expressed postsynaptically. Postsynaptic depolarization had no influence on firing patterns, action potential shape, or neuronal excitability. An increase in [Ca^2+^]_i_ in spinal lamina I neurons induces a non-Hebbian form of synaptic plasticity in spinal nociceptive pathways without affecting neuronal active and passive membrane properties.

## Summary

This study identified and characterized non-Hebbian type LTP in nociceptive pathways, a potential synaptic mechanism of secondary hyperalgesia.

## 1 Introduction

Primary hyperalgesia at the site of an injury or an inflammation may arise from nociceptor sensitization [18] and/or from amplified nociception in the central nervous system. A suggested central pain amplifier consists of activity-dependent long-term potentiation of synaptic strength (LTP) at C-fiber terminals in the superficial spinal dorsal horn [53]. We have demonstrated previously that LTP can be induced at synapses between primary afferent C-fibers and lamina I projection neurons of the superficial spinal dorsal horn by the application of conditioning electrical high-frequency stimulation (HFS) to the dorsal root at C-fiber strength. HFS-induced LTP requires activation of postsynaptic NMDA receptors and a rise in postsynaptic [Ca^2+^]_i_ [26]. According to Hebb’s postulate [19], induction of LTP requires coincident activation of the presynaptic and postsynaptic site, thereby leading to potentiation at activated synapses only. HFS-induced LTP at C-fiber synapses might therefore be regarded as Hebbian type. If LTP was expressed at stimulated synapses only, it would constitute a mechanism of primary hyperalgesia solely. Hyperalgesia typically also occurs, however, in the area outside the primary lesion where neither nociceptor activation nor peripheral sensitization occurs. The mechanisms underlying this secondary hyperalgesia are considered to be purely central. Much progress has been made in elucidating the neuronal mechanisms of secondary hyperalgesia [7], [53], [59], but some fundamental questions are still unresolved. For example, it is so far unknown how nociceptive input in one pathway triggers pain amplification in nearby, nonstimulated pathways, and how conditioning HFS leads to secondary hyperalgesia in humans [34], [35].

We hypothesize that conditioning HFS not only triggers LTP at stimulated synapses (Hebbian-type LTP), but also leads to LTP at nonstimulated synapses converging onto the same postsynaptic neurons (non-Hebbian LTP). We thus tested whether a rise in postsynaptic [Ca^2+^]_i_ may be sufficient for LTP induction at converging nonstimulated C-fiber synapses (ie, non-Hebbian form of LTP). An alternative or an additional mechanism of secondary hyperalgesia would be the enhanced membrane excitability of nociceptive neurons in spinal dorsal horn. This would likewise lead to enhanced nociception not only in stimulated pathways, but also in nonstimulated converging pathways [53]. We therefore also tested for intrinsic plasticity, ie, lasting modifications in membrane properties that affect the input–output function of neurons [12], [66].

In the present study we demonstrated that non-Hebbian LTP can be induced by patterned postsynaptic depolarizing stimuli, whereas intrinsic membrane properties remain stable.

## 2 Methods

### 2.1 Ethical approval

All experiments were in accordance with directive 2010/63/EU of the European Parliament and the council of the EU, and were approved by the Austrian Federal Ministry for Education, Science, and Culture.

### 2.2 Preparation of spinal cord slices

Male Sprague Dawley rats (17 to 26 days old) bred at Himberg and cared for by the Medical University of Vienna breeding facility were used for all experiments. Rats were decapitated under deep isoflurane anesthesia. The spinal cord was exposed by laminectomy and quickly removed into ice-cold incubation solution consisting of (in mM): NaCl 95, KCl 1.8, KH_2_PO_4_ 1.2, CaCl_2_ 0.5, MgSO_4_ 7, NaHCO_3_ 26, glucose 15, sucrose 50, oxygenated with 95% O_2_, 5% CO_2_; pH 7.4, measured osmolarity 305–320 mosmol · L^−1^. Transverse, 400–600-μm-thick slices with dorsal roots attached were cut with a vibrating microslicer (DTK-1000, Dosaka EM, Kyoto, Japan). Slices were incubated at 33 °C for at least half an hour and were oxygenated with carbogen (95% O_2_, 5% CO_2_). Slices were then stored in the same solution at room temperature (21–24 °C). A single slice was then transferred to the recording chamber, where it was continuously perfused at a rate of 2–3 mL · min^−1^ with oxygenated recording solution. The consistence of the recording solution was identical to the incubation solution except for (in mM): NaCl 127, CaCl_2_ 2.4, MgSO_4_ 1.3, sucrose 0. Recordings were performed at 32 ± 1 °C with a single-channel in-line solution heater (Supertech Ltd International GmbH, Pecs, Hungary).

### 2.3 Electrophysiological recordings

Superficial dorsal horn neurons were visualized with Dodt-infrared optics with a 40 × 0.80 NA water-immersion objective on an Olympus BX51WI upright microscope (Olympus Corporation, Tokyo, Japan). Neurons right beneath the dorsal white–gray matter border were considered as being lamina I neurons and used for the experiments. Standard whole-cell patch-clamp recording techniques were used in all experiments performed. Recorded neurons were investigated in voltage-clamp and current-clamp mode with an Axopatch 200B amplifier (Axon Instruments, Union City, CA, USA). Data were low-pass filtered at 10 kHz, amplified 2 times, and sampled at 10 kHz. The software package pCLAMP10 was used for data acquisition (Molecular Devices, Sunnyvale, CA, USA).

### 2.4 Induction of spinal LTP by depolarization

Only neurons with monosynaptic C-fiber input were tested for induction of synaptic plasticity in this study. EPSCs were classified as C-fiber evoked for conduction velocities below 0.5 m s^−1^. Monosynaptic C-fiber input was identified by the absence of failures in response to 10 stimuli given at 2 Hz, and a jitter in response latencies of less than 2 ms. Patch pipettes were made from borosilicate glass on a horizontal micropipette puller (P-87, Sutter Instruments, Novato, CA, USA) and revealed an impedance of 2–5 MΩ when filled with the pipette solution consisting of (in mM): K-gluconate 120, KCl 20, MgCl_2_ 2, Na_2_ATP 2, Na-GTP 0.5, HEPES 20, Na_4_EGTA 0.5, pH 7.28 with KOH, measured osmolarity ∼300 mosmol L^−1^. In some of the experiments, the Ca^2+^ chelator BAPTA (20 mM; Sigma) was added to the pipette solution consisting of (in mM): K-gluconate 80, KCl 20, MgCl_2_ 2, Na_2_ATP 2, NaGTP 0.5, HEPES 20, Na_4_EGTA 0.5, pH 7.28 with KOH, measured osmolarity ∼300 mosmol L^−1^. In some experiments, the selective NMDA receptor blocker MK-801 [(+)-5-methyl-10,11-dihydro-5*H*-dibenzo*[a,d]*cyclo-hepten-5,10-imine maleate)] (1 mM; Tocris) was added to the standard pipette solution to block postsynaptic NMDA receptors. Nifedipine (50 μM; Sigma) or ryanodine (20 μM; Tocris) were added to the oxygenized superfusion solution at least 15 to 20 min before the conditioning depolarization. Stock solutions were prepared by dissolving the drugs in dimethylsulfoxide (DMSO; Sigma) and stored in aliquots at −20 °C. Aluminium foil was used to cover the tubes in all experiments with nifedipine because of its light sensitivity.

Dorsal roots were stimulated through a suction electrode with an isolated current stimulator (World Precision Instruments, Sarasota, FL, USA) for the recording of primary afferent evoked excitatory postsynaptic currents (EPSCs). Test pulses of 0.1 ms were applied at intervals of 30 s. The stimulation intensity of the test pulse was kept constant over the recording period. For the assessment of the paired pulse ratio (PPR), a second stimulation was applied with an interstimulus interval of 300 ms between the test pulses; a paired pulse depression was observed in all the EPSC recordings performed.

For the induction of synaptic LTP, different conditioning stimuli were used. In experiments where paired high-frequency stimulation (HFS) was applied, the dorsal root fibers were stimulated by 3 trains of 10 mA pulses provided at 200 Hz for 1 s at 10 s intervals. Neurons were simultaneously depolarized to about −30 mV. For the remaining experiments, postsynaptic depolarization without any presynaptic stimulation was used as the conditioning stimulation. Neurons were depolarized to about −30 mV in the current-clamp mode using different stimulation patterns: steady depolarization for 22 s, 6 depolarizing pulses for 1 s, 12 depolarizing pulses for 1 s, or 6 depolarizing pulses for 1 s combined with 22 s steady depolarization with 13 s interstimulus interval without current injection in between. For one series of experiments, a HFS surrogate was used as the conditioning stimulation. To identify a HFS surrogate, we first recorded the membrane potentials during the application of HFS from several neurons expressing LTP, and used a typical pattern of membrane potential fluctuation as HFS surrogate.

### 2.5 Ca^2+^ imaging

For Ca^2+^ imaging experiments, the internal pipette solution was composed of (in mM): K-gluconate 120, KCl 20, MgCl_2_ 2, HEPES 20, Na_2_ATP 2, NaGTP 0.5, fura-2 pentapotassium salt 0.25 (Fluka), pH 7.28 adjusted with KOH; measured osmolarity ∼300 mosmol L^−1^. Lamina I neurons were loaded for at least 8 min with the fluorescent dye fura-2 via the patch pipette and illuminated with a monochromator. The monochromator extracts the desired wavelengths from white light using an optical grid in about 1 to 2 ms. Digital fluorescence images were obtained using consecutive exposures to 340 and 380 nm. The dichroic filter 410 nm and the long-pass excitation filter 440 nm from Till Photonics GmbH were used. The emission wavelength was 510 nm. Images were obtained at 2–5 Hz with a cooled CCD camera (TILLvisION Imaging system, TILL Photonics GmbH, Munich, Germany; Q-imaging, Surrey, BC, Canada). Kinetics of intracellular somatic Ca^2+^ signals were calculated off-line by ratiometric fluorescence (F_340_/F_380_).

### 2.6 Recording of intrinsic plasticity

The membrane potential measured in the absence of any current injection was considered as the resting membrane potential (RMP). Only neurons revealing a resting membrane potential more negative than −45 mV were studied further. For the investigation of the action potential (AP) threshold, neurons were held at −80 mV in the voltage-clamp configuration and depolarized in 2 mV steps until a strong Na^+^ current underlying an AP was elicited. The difference between the resting membrane potential and AP threshold (ΔV) was calculated for every neuron and considered as a contributing parameter of neuronal excitability. For the determination of the firing pattern, depolarizing current was injected 6 times for 1 s with increasing strength and 10 s interstimulus intervals each, starting from a membrane potential of −75 ± 5 mV. The increments of current injections were kept constant in each recording and ranged from 20 pA to 160 pA. Protocols for the determination of the RMP, AP threshold, and firing pattern were repeated every 10 min to compare the neuronal properties before and after the conditioning stimuli.

Membrane resistance, series resistance, holding current, and capacitance were measured by a hyperpolarizing voltage step from −70 mV to −80 mV and monitored throughout the whole experiment. Series resistance ranged from 8 to 20 MΩ. Neurons with changes in the series resistance of over 35% were excluded from further analysis. Offset and capacity were corrected at the beginning of each experiment. Recordings were discarded if the offset exceeded ±3.5 mV at the end of each experiment. No correction for the liquid junction potential was made.

### 2.7 Data analysis

Data were analyzed off-line using Clampfit 10 (Molecular Devices, Sunnyvale, CA, USA) and SigmaPlot 11 (Systat Software GmbH, Erkrath, Germany). Values are presented as means ±1 standard error of the mean (SEM). For the quantification of synaptic strength, the peak amplitude of the evoked EPSC was measured. The mean amplitudes of 4 EPSCs before the conditioning stimuli served as control. LTP was defined as a significant increase in the EPSC amplitudes after the conditioning stimuli for at least 30 min. Only a potentiation above 120% of normalized baseline values was considered as LTP. One-way repeated-measures (RM) ANOVA with Bonferroni adjustment or the nonparametric RM ANOVA on ranks with Dunnett’s adjustment was performed to test for potentiation statistically. Effects of drugs were tested by Fisher’s exact test.

PPR was determined by dividing the second EPSC amplitude by the first EPSC amplitude. Squared coefficient of variation was calculated from the first EPSC amplitude every 5 min and normalized to the baseline values (CV^−2 ^= mean^2^/variance) [16]. The mean of 4 PPR and CV^−2^ values before the conditioning stimuli served as control values.

For the determination of the AP width, AP height, and the afterhyperpolarization (AHP) amplitude, the first AP elicited upon positive current injection for the determination of the firing pattern was used. Values were measured starting from the base of the AP.

Baseline values of the RMP, AP threshold, ΔV, membrane resistance, AP height, AP width, and AHP amplitude (Pre) were compared to the data acquired 30 min later (Post) for the control group without depolarization and the depolarized group, respectively. Conditioning depolarizing stimulation (CS) was performed at time point 0 min after the recording of the baseline values. Statistical analysis was performed by Student’s paired *t* test or the nonparametric Wilcoxon signed rank test (Pre–Post comparison), by Student’s unpaired *t* test or the nonparametric Mann-Whitney rank sum test (comparison control, CS), or with 1-way ANOVA or the Kruskal-Wallis ANOVA on ranks for nonnormally distributed data (comparison control, LTP, no LTP). *P* < .05 was considered statistically significant.

## 3 Results

### 3.1 The pattern of postsynaptic depolarization determines the effects on synaptic strength

**Fig. 1:**
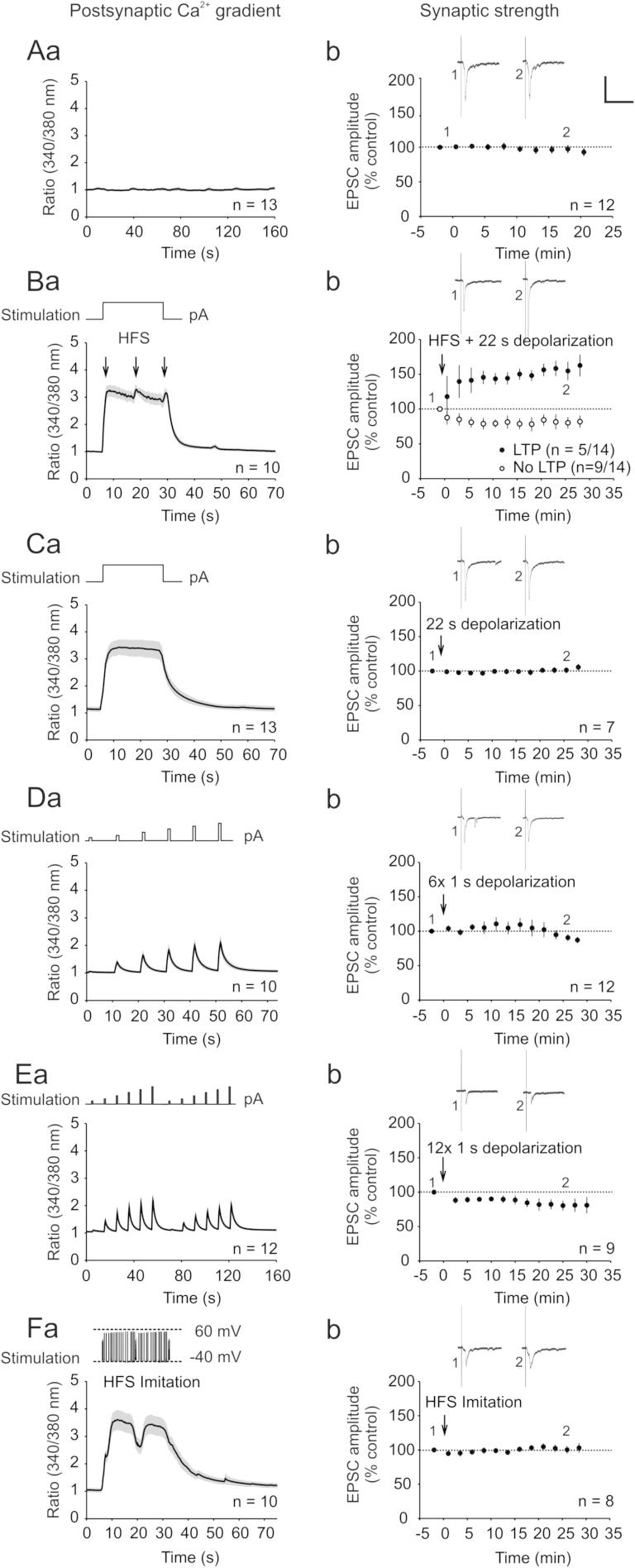
Impact of various conditioning stimuli on Ca^2+^ signals and synaptic strength. (Aa–Fa) Ca^2+^ signals elicited with different conditioning stimulation patterns are plotted against the time (s). Black lines represent mean values; gray areas, SEM. Pattern of depolarizing current injection/membrane potential is shown at the top of the graphs in (Ba–Fa), respectively. Arrows in (Ba) indicate HFS stimulation to attached dorsal root. (Ab–Fb) In all graphs, monosynaptic C-fiber-evoked EPSC amplitudes were normalized to prestimulation values (dotted line) and plotted against time (min). One dot represents the mean of 4 to 5 EPSC amplitudes, respectively; in (Bb–Fb), conditioning stimuli were applied at time point 0 min (arrows). Insets show original traces of C-fiber-evoked EPSCs at indicated time points; calibration bars indicate 100 ms and 400 pA. (Aa) Control recordings without any stimulation show a stable Ca^2+^ signal over the observation period of 160 s. (Ab) C-fiber EPSC amplitudes stay constant over a recording time of 25 min. (B) Paired HFS consisting of HFS applied 3 times at 200 Hz to the dorsal root with 10 s interstimulus interval, respectively, and of postsynaptic depolarization to −30 mV simultaneously increased the postsynaptic Ca^2+^ signal (Ba) and induced synaptic LTP in 5 of 14 neurons recorded (filled cycles). No potentiation was observed in the remaining 9 neurons (open cycles) (Bb). (C) Sustained postsynaptic depolarization for 22 s clearly increased the postsynaptic Ca^2+^ signal for the duration of the stimulation (Ca) but did not affect the strength of synaptic transmission (Cb). (D) Six postsynaptic depolarizing pulses for 1 s with increasing intensities and with 10 s interstimulus intervals each stepwise increased the Ca^2+^ signal (Da) but did not induce LTP in any of the 8 neurons tested (Db). (E) Stimulation protocol in (D) applied twice did not cause any change in the strength of synaptic transmission either (Eb). (F) Membrane potential of one neuron expressing LTP after HFS was applied as the conditioning stimulation (HFS surrogate). No LTP was observed in any of the neurons recorded (Fb).

We first confirmed that spinal LTP could be induced under the given experimental conditions by applying a standard protocol for LTP induction. HFS of afferents in the attached ipsilateral dorsal root at C-fiber intensity and simultaneous postsynaptic depolarization (paired HFS) induced a strong postsynaptic Ca^2+^ rise with small peaks during conditioning stimulation (*n* = 10; highest Ca^2+^ signal ratio (340/380) 3.28 ± 0.16; *P* ⩽ .001 by Student’s paired *t* test; Fig. 1Ba). Paired HFS was followed by a significant, lasting increase in the C-fiber evoked EPSC amplitude in 5 of 14 spinal lamina I neurons recorded (to 163 ± 16% of control 30 min after HFS, *P* < 0.05 by 1-way RM ANOVA; Fig. 1Bb). Control experiments without any conditioning stimulation confirmed the stability of baseline Ca^2+^ levels throughout the recording period (*n* = 13; highest Ca^2+^ signal ratio (340/380) 1.15 ± 0.11; *P* > 0.05 by Wilcoxon signed rank test, Fig. 1Aa) and EPSC amplitudes (*n* = 12; 97 ± 6% of control at time point 20 min, *P* > 0.05, RM ANOVA on Ranks; Fig. 1Ab).

To test whether postsynaptic depolarization in the absence of any presynaptic conditioning stimulation is sufficient for LTP induction, we applied several depolarizing stimulation patterns to spinal lamina I neurons as conditioning stimuli. We recorded intracellular Ca^2+^ signals and compared the amplitudes of EPSCs evoked by electrical stimulation of dorsal horn afferents before and after conditioning stimulation. First, we depolarized the neurons to −30 mV for 22 s, which is identical to the postsynaptic depolarization protocol during paired HFS. This stimulation led to a Ca^2+^ kinetic quite similar to that of the paired HFS but lacking the peaks (*n* = 13; highest Ca^2+^ signal ratio (340/380) 3.43 ± 0.28; *P* ⩽ 0.001 by Student’s paired *t* test; Fig. 1Ca). LTP was not induced in any of the neurons tested after steady postsynaptic depolarization for 22 s. EPSC amplitudes stayed stable at 106 ± 5% of control after 30 min (*n* = 7; *P* > 0.05 by 1-way RM ANOVA, Fig. 1Cb).

We next asked whether the pulsed spikes observed during the HFS application were indispensable for the induction of synaptic LTP. We thus applied a pulsed conditioning stimulus. Neurons were depolarized 6 times by increasing current injection at 10 s interstimulus intervals. This stimulation protocol elicited regular Ca^2+^ spikes similar to the Ca^2+^ peaks observed during paired HFS (*n* = 10; highest Ca^2+^ signal ratio (340/380) 2.10 ± 0.24; *P* ⩽ 0.05 by Wilcoxon signed rank test; Fig. 1Da). Synaptic transmission was, however, not affected by this pulsed stimulation protocol (*n* = 12; 87 ± 5% of control after 30.5 min, *P* > 0.05 by RM ANOVA on ranks, Fig. 1Db). To exclude that stimulation with 6 pulses may have been below the threshold for LTP induction, we next applied the same protocol twice. Twelve depolarizing pulses led to regular Ca^2+^ spikes (*n* = 12; highest Ca^2+^ signal ratio (340/380) 2.20 ± 0.20; *P* ⩽ 0.001 by Wilcoxon signed rank test; Fig. 1Ea). LTP was not induced in any of the neurons recorded. EPSC amplitudes stayed at 81 ± 10% of control at 30 min (*n* = 9; *P* > 0.05 by RM ANOVA on ranks; Fig. 1Eb).

We then asked whether neurons that expressed LTP display specific patterns of membrane potential fluctuation during paired HFS that are essential for the induction of synaptic potentiation. We recorded the membrane potentials during the application of HFS from several neurons expressing LTP, and we used a typical pattern of membrane potential fluctuation for conditioning stimulation (surrogate HFS) in other lamina I neurons. During surrogate HFS, Ca^2+^ signals significantly rose above baseline (*n* = 10; highest Ca^2+^ signal ratio (340/380) 3.61 ± 0.36; *P* ⩽ 0.001 by paired *t* test; Fig. 1Fa) but failed to induce LTP in any of the 8 lamina I neurons tested (103 ± 7% of control after 30.5 min; *P* > 0.05 by RM ANOVA on ranks; Fig. 1Fb). Thus, sustained or pulsed depolarization alone was ineffective for LTP induction. Collectively, our results indicate that LTP cannot be induced by postsynaptic depolarization leading to a rise in [Ca^2+^]_i_, which mimics the temporal patterns of [Ca^2+^]_i_ during paired HFS that induced LTP.

**Fig. 2:**
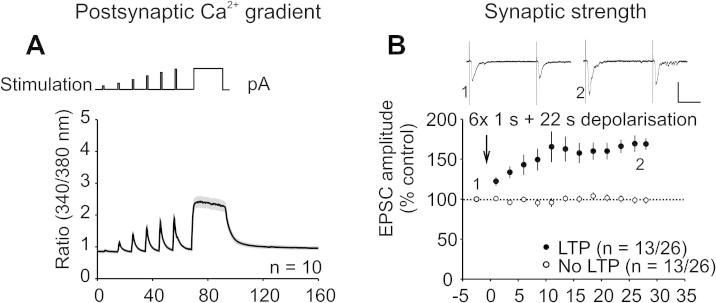
LTP induction after pulsed and sustained postsynaptic depolarization. (A) Ca^2+^ signals during postsynaptic depolarization are plotted vs time (s). (B) Averaged time course of monosynaptic C-fiber evoked EPSCs before and after application of the conditioning stimulus (arrow). Values were normalized to baseline values (dotted line). Filled circles represent neurons showing potentiation; open cycles, neurons showing no reaction after the conditioning stimulation. Insets show original traces of C-fiber evoked EPSCs at indicated time points; calibration bars indicate 100 ms and 400 pA. Combination of 6 pulsed depolarization for 1 s and sustained depolarization for 22 s with an interstimulus interval of 13 s at resting membrane potential in between induced a marked elevation in Ca^2+^ signal in all neurons tested (A) and induced LTP in 50% of the neurons tested (B).

We next asked whether a pattern of postsynaptic Ca^2+^ rise can be identified that is capable of inducing LTP at C-fiber synapses, ie, whether spinal lamina I neurons can express non-Hebbian types of LTP. Repetitive depolarizing stimuli can induce priming in intracellular Ca^2+^ stores leading to larger intracellular Ca^2+^-induced Ca^2+^ waves in primed neurons than in neurons where no priming has been performed [23]. Furthermore, priming protocols using repetitive depolarization have been reported to be more efficient for inducing intracellular Ca^2+^ release than protocols using sustained depolarizing stimuli [23]. We thus combined the pulsed stimulation and the sustained depolarization protocol for 22 s (*n* = 10; highest Ca^2+^ signal ratio (340/380) 2.42 ± 0.18; *P* ⩽ 0.001 by Student’s paired *t* test; Fig. 2Aa). This temporal pattern of membrane depolarization is reminiscent of plateau potentials observed in a subgroup of spinal dorsal horn neurons upon repetitive action potential discharges. This surrogate plateau potential now induced LTP in 13 of 26 neurons tested to 169 ± 7% of control after 30 min (*P* < 0.001 by RM ANOVA on ranks, Dunnett’s post hoc test; Fig. 2B).

**Fig. 3:**
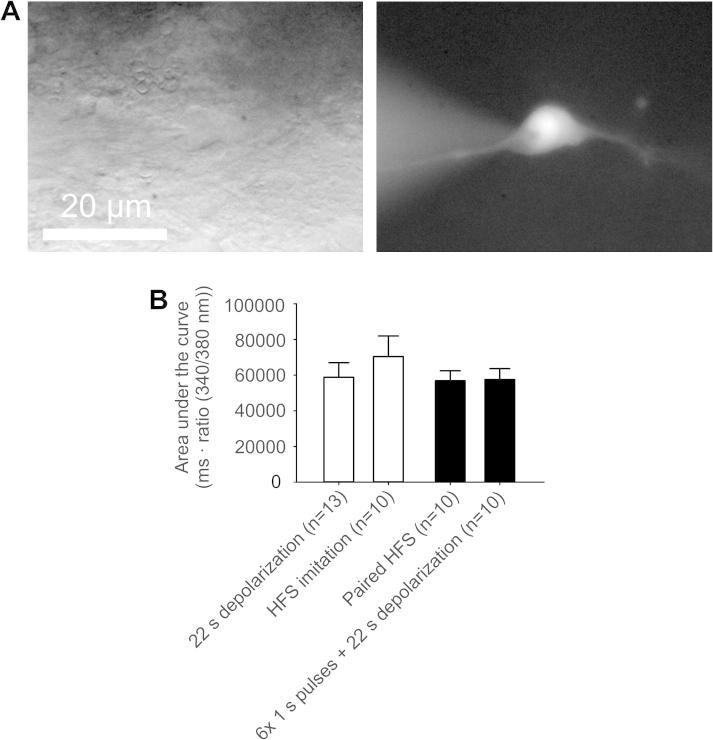
Comparison of the area under the curves of different Ca^2+^ kinetics. (A) Transmission and fluorescence image of a lamina I neuron labeled with the Ca^2+^-sensitive dye fura-2. (B) Area under the curves of Ca^2+^ kinetics elicited by LTP inducing stimulations (black bars) were compared with the area under the curves of Ca^2+^ signals induced by stimuli leading to no potentiation (open bars). There were no significant differences in the magnitude of Ca^2+^ levels after stimulation protocols that induced LTP and those that did not induce LTP (*P* > .05 by Kruskal-Wallis 1-way ANOVA on ranks).

We also asked whether stimulation protocols that induced LTP elicited significantly higher Ca^2+^ signals than stimulation protocols that failed to induce LTP. When we quantitatively compared the area under the curves of the Ca^2+^ kinetics after the different stimulation protocols, no significant differences were observed between the Ca^2+^ levels (*P* > 0.05 for all comparisons by Kruskal-Wallis 1-way ANOVA on ranks; Fig. 3B). This indicates that the magnitude of the Ca^2+^ signal does not correlate with the induction of synaptic plasticity, suggesting that the temporal and/or spatial pattern of the Ca^2+^ gradient are relevant parameters.

### 3.2 Characterization of depolarization-induced LTP

For all subsequent experiments, the surrogate plateau potential (Fig. 2Aa) was used because it induced non-Hebbian LTP in a significant proportion of neurons.

**Fig. 4:**
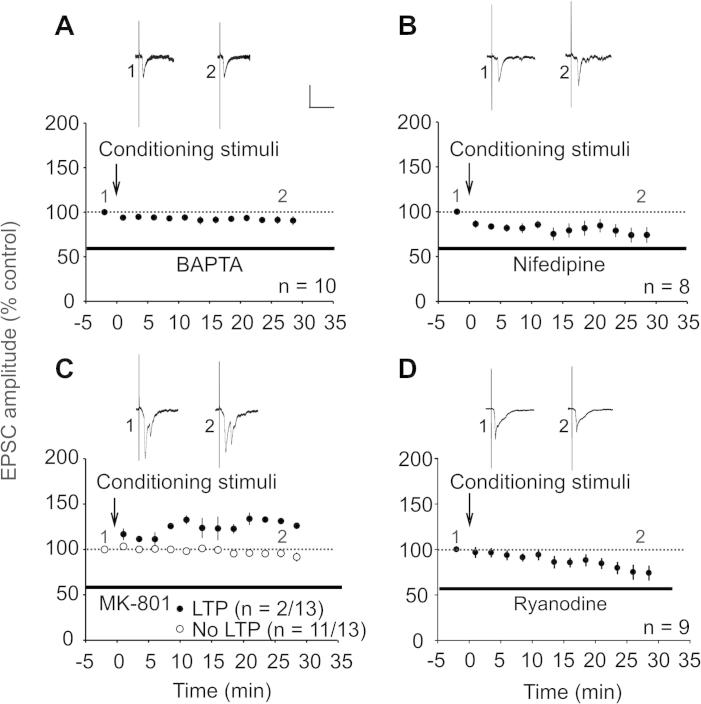
Pharmacological characterization of non-Hebbian LTP. Averaged time course of monosynaptic C-fiber evoked EPSCs before and after application of the conditioning stimuli (arrow). Values were normalized to baseline values (dotted line). Insets show original traces of C-fiber-evoked EPSCs at indicated time points; calibration bars indicate 100 ms and 400 pA. Six depolarizing pulses for 1 s followed by sustained depolarization for 22 s (Fig. 2Aa) were used as LTP conditioning stimulation (CS) in this and all other subsequent figures if not otherwise stated. (A) Application of 20 mM BAPTA into the pipette solution blocked LTP induction in all 10 neurons tested (*P* = .006 when compared to LTP after CS in the absence of BAPTA; Fisher’s exact test). (B) Blocking L-type VGCCs with 50 μM nifedipine added to the bath solution inhibited induction of synaptic LTP in 8 neurons tested (*P* = .013 when compared to LTP after CS in the absence of nifedipine, Fisher’s exact test). (C) Application of postsynaptic NMDA receptor blocker MK-801 into the pipette solution significantly reduced the induction rate of LTP (*n* = 2 of 13; *P* = .045 when compared to LTP induced after CS in the absence of MK-801, Fisher’s exact test). (D) CS in the presence of 20 μM ryanodine blocked LTP in all 9 neurons tested (*P* = .013 when compared to LTP induced after CS in the absence of ryanodine, Fisher’s exact test).

To test whether depolarization-induced LTP depends on a rise in postsynaptic Ca^2+^, we added the Ca^2+^ chelator BAPTA to the pipette solution. This completely blocked potentiation in all neurons tested (*n* = 10; 91 ± 5% of control after 30 min; *P* > 0.05 by 1-way RM ANOVA; *P* < .05 by Fisher’s exact test compared to stimulation without BAPTA; Fig. 4A). L-type VGCCs are activated in spinal lamina I neurons upon postsynaptic depolarization [20]. We therefore tested whether L-type VGCCs need to be activated to induce LTP. We used nifedipine in the bath solution to block L-type VGCC, which might, however, also have nonspecific effects such as blocking voltage-dependent Na^+^ channels [1], [22], [65]. To test whether nifedipine blocks voltage-dependent Na^+^ channels, we compared the number of action potentials during conditioning postsynaptic depolarization with and without nifedipine. There was no significant reduction in the number of APs in the presence of nifedipine (data not shown), suggesting that sodium channels required for action potential discharges were not blocked. In the presence of nifedipine, LTP could not be induced in any of the neurons recorded (*n* = 8; 74 ± 9% of control after 30 min; *P* > 0.05 by RM ANOVA on ranks; *P* < 0.05 by Fisher’s exact test compared to stimulation in the absence of nifedipine; Fig. 4B). Thus, activation of L-type VGCCs seems to be necessary for LTP induction by postsynaptic depolarization. We also examined the role of postsynaptic NMDA receptors by adding the selective NMDA receptor blocker MK-801 to the pipette solution. LTP was induced under these conditions in 2 of 13 neurons (126 ± 2% of control after 30 min; Fig. 4C). Fisher’s exact test indicated no significant difference compared to the control group (no stimulation), and significantly less LTP compared to the group using the same conditioning depolarization in the absence of MK-801. Taken together, the data suggest that LTP induction by postsynaptic depolarization may also involve the activation of postsynaptic NMDA receptors.

To test the hypothesis that repetitive depolarization leads to priming of internal Ca^2+^ stores via Ca^2+^-induced Ca^2+^ release, we used a high concentration of ryanodine (20 μM), which blocks Ca^2+^ release from ryanodine sensitive stores. We have previously demonstrated that at this concentration ryanodine has no effects on baseline synaptic transmission [21]. Using ryanodine, no LTP was induced in any of the 9 neurons tested (74 ± 8% of control after 30 min; *P* < 0.05 by Fisher’s exact test compared to stimulation in the absence of ryanodine). Six of 9 neurons instead exhibited a significant decrease in the EPSC amplitudes 30 min after conditioning stimulation (*P* < 0.001 by RM ANOVA; Fig. 4D).

**Fig. 5:**
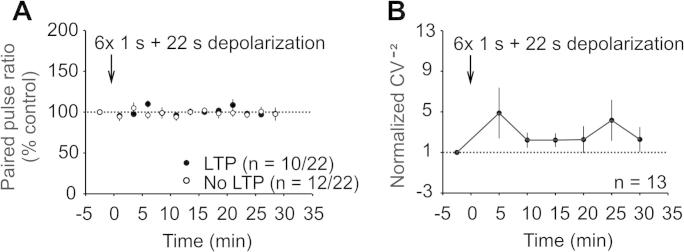
Paired pulse ratio (PPR) and coefficient of variation. (A) PPRs of neurons showing significant potentiation did not differ significantly from PPRs of neurons that did not potentiate 30 min after the conditioning stimulation (*P* = 0.992 by Student’s unpaired *t* test). (B) Normalized and squared coefficient of variation (CV^−2^) was not significantly changed in all time points recorded (*P* = 0.157 by RM ANOVA on ranks).

We next asked if LTP induced by postsynaptic depolarization is expressed pre- or postsynaptically. Conditioning stimulation did not affect PPR, either in neurons that expressed LTP (*n* = 10; 98 ± 4% of control after 6 times 1 s + 22 s depolarization; after 30 min) or in neurons that did not express LTP (*n* = 12; 98 ± 8% of control after 6 times 1 s + 22 s depolarization; after 30 min, respectively; *P* > 0.05 by Student’s unpaired *t* test, respectively; Fig. 5A). Likewise, squared and normalized coefficient of variation (CV^−2^) indicated no significant difference after conditioning stimulation compared to baseline values (*n* = 13; *P* > .05 by 1-way RM ANOVA on ranks; Fig. 5B). The stability of both PPR and CV^−2^ suggests that the expression of the non-Hebbian type of LTP is likely postsynaptic in nature.

### 3.3 Membrane properties of spinal lamina I neurons after postsynaptic depolarization

Intrinsic membrane properties of lamina I neurons were assessed before and after conditioning depolarizing stimulation using the same surrogate plateau potential protocol that induced non-Hebbian LTP. EPSCs were recorded throughout the experiment to also test for changes in synaptic strength in the same neurons (Fig. 2B).

**Fig. 6:**
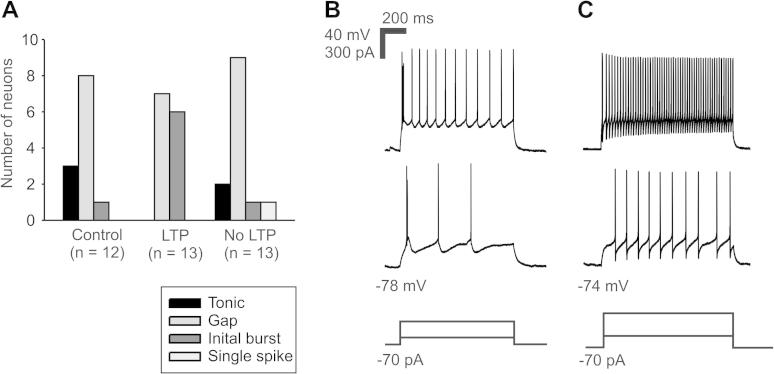
Firing patterns of lamina I neurons expressing non-Hebbian LTP. (A) Neurons expressing synaptic LTP after a conditioning stimulus exhibited either the gap or the initial burst firing pattern, whereas neurons showing no potentiation fired mostly in the gap firing pattern, or rarely in tonic, initial burst, or single spike pattern. The gap firing pattern was the most common firing pattern in all of the groups. (B) Typical example of initial burst firing pattern showing 2 or more bursts of action potentials at the beginning of the positive current injection. (C) Representative example of gap firing pattern with the characteristic long first interspike interval. Bottom traces show depolarizing current injections superimposed.

Neurons were grouped as LTP-expressing and non-LTP-expressing neurons, then further classified into 4 categories according to their firing patterns. Neurons expressing non-Hebbian LTP exhibited either the gap firing pattern or the initial burst firing pattern (Fig. 6B, C). The group of neurons expressing no LTP predominantly displayed the gap firing pattern and rarely tonic firing pattern, initial burst firing pattern, or single spike firing pattern. Neurons of the control group exhibited mostly gap, and in some cases tonic or initial burst, firing patterns (Fig. 6A). Conditioning depolarization did not change the firing pattern in any group of neurons (data not shown).

We compared a measure of neuronal membrane excitability (ΔV), resting membrane potential (RMP), and action potential threshold (AP threshold) during the baseline period (Pre) and 30 min after conditioning stimulation (Post). Conditioning stimulation did not change the RMP, the AP threshold, or ΔV in any of the groups tested. RMP of neurons expressing LTP were not less negative 20 or 30 min after conditioning stimulation (*n* = 10; Pre: −57 ± 4; Post: −51 ± 2) than neurons of the control group 20 or 30 min after baseline recordings (*n* = 9; Pre: −55 ± 1; Post: −58 ± 4), providing no evidence for any increase in excitability in synaptically potentiated neurons (Table 1). No change in the AP threshold or in ΔV could be observed in any of the groups tested (*n* = 9 in the control group, *n* = 16 in the CS group; *P* > .05 by Student’s paired *t* test or the nonparametric Wilcoxon signed rank test) or between the groups (*P* > .05 by Kruskal-Willis ANOVA on ranks). Likewise, no changes in active or passive membrane properties were observed when neurons were further subdivided by the expression of LTP (*n* = 10 in LTP-expressing group, *n* = 6 in the group expressing no LTP; *P* > .05 by Student’s paired *t* test or the nonparametric Wilcoxon signed rank test for comparison between Pre and Post; *P* > .05 by 1-way ANOVA or the nonparametric Kruskal-Willis ANOVA on ranks for comparisons between the groups; data summarized in Table 1). Conditioning depolarization had no effect on AP height or width, or on membrane resistance compared to control (Table 1).

**Table 1:** Summary of intrinsic membrane properties in spinal lamina I neurons^a^.

Characteristic	Control (*n* = 9)	CS (LTP + no LTP) (*n* = 16)	LTP (*n* = 10)	No LTP (*n* = 6)
*RMP, mV*				
Pre	−55 ± 1	−55 ± 3	−57 ± 4	−52 ± 2
Post	−58 ± 4	−52 ± 1	−51 ± 2	−54 ± 1

*Action potential threshold, mV*				
Pre	−34 ± 2	−37 ± 2	−40 ± 3	−33 ± 3
Post	−33 ± 2	−37 ± 2	−39 ± 3	−33 ± 4

*RMP* − *action potential threshold, ΔV, mV*				
Pre	21 ± 2	18 ± 4	17 ± 6	19 ± 2
Post	25 ± 5	15 ± 3	12 ± 3	21 ± 4

*Action potential height, mV*				
Pre	87 ± 5	89 ± 3	89 ± 4	88 ± 5
Post	77 ± 5⁎	85 ± 3⁎	87 ± 3	83 ± 6⁎

*Action potential width at base, ms*				
Pre	1.5 ± 0.2	1.4 ± 0.1	1.5 ± 0.1	1.3 ± 0.1
Post	1.5 ± 0.2	1.4 ± 0.1	1.4 ± 0.1	1.3 ± 0.1

*Posthyperpolarization amplitude, mV*				
Pre	−30 ± 3	−30 ± 3	−27 ± 3	−35 ± 4
Post	−33 ± 3⁎	−30 ± 3	−26 ± 3⁎	−37 ± 5

*Membrane resistance, MΩ*				
Pre	712 ± 89	601 ± 131	592 ± 151	616 ± 240
Post	380 ± 83⁎	346 ± 77⁎	408 ± 119	243 ± 37⁎

CS, conditioning depolarizing stimulation; LTP, long-term potentiation; RMP, resting membrane potential.

a Data are presented as mean ± 1 standard error of the mean. No statistically significant difference was observed in any of the stimulated groups compared to the control group, nor between any of the depolarized groups, respectively.

⁎ *P* < 0.05 compared to baseline values (Pre).

## 4 Discussion

Hebbian-type LTP has been demonstrated by numerous studies in different regions of the central nervous system including the dorsal horn of the spinal cord [26], [27], [48]. In striking contrast, information about non-Hebbian LTP is scarce [31], [55], [63]. Here we demonstrate that spinal lamina I neurons express a non-Hebbian type of LTP after a rise in [Ca^2+^]_i_ due to postsynaptic depolarization. Intrinsic membrane properties were, in contrast, not affected in these lamina I neurons.

### 4.1 Non-Hebbian LTP at C-fiber synapses in spinal dorsal horn neurons

Various models of neuropathic and nociceptive pain are associated with secondary hyperalgesia [46], [62] and with elevated levels of [Ca^2+^]_i_ in spinal superficial dorsal horn [5], [8], [32], [45]. Until now, it has, however, never been demonstrated directly that a rise in postsynaptic [Ca^2+^]_i_ is sufficient for the generation of non-Hebbian type of synaptic plasticity in nociceptive pathways. Non-Hebbian type LTP between primary afferent C-fibers and spinal lamina I neurons constitutes a potential mechanism of secondary hyperalgesia.

In a recent study [14], we induced LTP in the absence of any conditioning presynaptic stimulation by abrupt opioid withdrawal. At first glance, opioid-withdrawal LTP seems to be non-Hebbian because it illustrates some parallels with the presently identified LTP, ie, the independence from intentional presynaptic stimulation and the requirement of a postsynaptic Ca^2+^ rise and of NMDA receptor activation. Furthermore, the stability of the PPR as well as the CV^−2^ suggests that both forms of LTP are expressed postsynaptically, without requiring any retrograde messenger. Upon withdrawal, opioids may, however, enhance glutamate release [29], [58]. Hence, the possibility of pre- and postsynaptic costimulation to induce opioid-withdrawal LTP, ie, a Hebbian-type LTP, cannot be fully excluded. In the present study, we provide the first direct evidence for non-Hebbian LTP at synapses in the spinal dorsal horn. Our data demonstrate LTP induction by a surrogate plateau potential protocol consisting of pulsed depolarization followed by a continuous depolarization. Synaptic inputs generating plateau potentials may arise from rhythmic, bursting discharges with on and off phases in sciatic nerve axons, eg, after chronic nerve injury [13], [57]. A rhythmic discharge pattern may be caused by DRG membrane potential oscillations due to intermittent activation of Ca^2+^-dependent K^+^ channels [38]. Similar to the presently identified non-Hebbian LTP, plateau potentials also require activation of L-type VGCCs [40], [50] and NMDA receptors [56].

Spinal lamina I neurons expressing non-Hebbian LTP fired action potentials in the gap firing pattern or the initial burst firing pattern. This result is in accordance with our previous studies demonstrating that LTP is preferentially induced in lamina I neurons projecting to the parabrachial region [26] or to the periaqueductal gray in the brain [27], which predominantly exhibit the gap firing pattern and the bursting firing pattern [49].

We hypothesize that Hebbian LTP at C-fiber synapses is a potential mechanism of primary hyperalgesia, while non-Hebbian LTP may lead to secondary hyperalgesia. Another functional consequence of non-Hebbian LTP could be mechanical allodynia, if synaptic transmission of low-threshold mechanosensitive C- or Aβ-fibers would be potentiated as well. It seems possible that non-Hebbian LTP is induced under some forms of neuropathy. In animal models of neuropathy, diverse forms of neuronal plasticity have been found in the superficial dorsal horn that could lead to elevated levels of postsynaptic [Ca^2+^]_i_—decrease in EPSC thresholds [36], decrease in mIPSC frequency [41], elevation in mEPSC frequency [28], and decrease in glutamate transporter expression [42]—suggesting an increased and prolonged glutamate spillover to dorsal horn neurons. Inhibition of glutamate transporters has further been demonstrated to increase neuronal excitability [61]. All these changes enhance neuronal excitation and might increase the frequency and/or the magnitude of intracellular Ca^2+^ waves in superficial dorsal horn neurons and thus possibly facilitate the induction of presently described non-Hebbian LTP.

Non-Hebbian LTP in spinal lamina I neurons depended on a rise in postsynaptic [Ca^2+^]_i_ and was blocked by nifedipine, suggesting that the activation of L-type VGCCs was necessary. The presently used nifedipine concentration might, however, also have additional effects, such as the blockade of sodium channels or the facilitation of neurotransmitter release [1], [22], [65]. In the present study the number of action potentials in the presence of nifedipine was not significantly smaller than under control conditions suggesting no substantial blockade of voltage-dependent sodium channels that are required for action potential generation. Strong sustained depolarizing stimuli gradually inactivate VGCCs [25], [52]. This may explain why sustained depolarizing protocols failed to induce non-Hebbian LTP. The staged, patterned depolarization leading to non-Hebbian LTP suggests that priming of signaling pathways may be involved, ie, an activation of signal transduction pathways that consequently regulate VGCCs by Ca^2+^-dependent mechanisms. It has, for example, been demonstrated that opening of L-type VGCC at the soma leads to the activation of the adenosine-3′,5′-phosphate (cAMP) pathway including activation of Ca^2+^/calmodulin-dependent protein kinase II (CaMKII). This activates nuclear gene transcription [4] which enhances L-type VGCC activity [6]. We suggest that the initial rise in Ca^2+^ triggers Ca^2+^-dependent Ca^2+^ release from ryanodine receptor-sensitive Ca^2+^ stores, which consequently activates intracellular mechanisms indispensable for the induction of non-Hebbian LTP. This is in line with our finding that non-Hebbian LTP was fully blocked by a ryanodine receptor antagonist.

### 4.2 The role of NMDA receptors for the induction of non-Hebbian type of LTP

Many forms of spinal pain amplification including the induction of some types of secondary hyperalgesia require activation of NMDA receptors [9], [60], [67]. There are, however, reports of other types of secondary hyperalgesia that are independent of NMDA receptor activation [30], [44], [47]. NMDA receptors are classically associated with Hebbian-type synaptic plasticity, as activation of NMDA receptors requires both binding of glutamate, as well as postsynaptic depolarization to remove the Mg^2+^ block of the NMDA receptors [39], [43]. The Hebbian coactivation of the presynaptic and postsynaptic site then leads to Ca^2+^ influx through NMDA receptor channels. Accordingly, most previously described forms of non-Hebbian LTP are independent of any activation of NMDA receptors [10], [31], [37]. Interestingly, our study revealed that induction of non-Hebbian LTP at C-fiber synapses with spinal lamina I neurons requires activation of NMDA receptors. Possibly ambient glutamate in the extracellular fluid is sufficient to bind to and to activate NMDA receptors upon depolarization. Ambient glutamate may originate from spontaneous glutamate release, eg, from spinal dorsal horn interneurons, from afferent fibers, as well as from descending neurons [64]. Ambient glutamate in the extracellular space has indeed been demonstrated to be sufficient to activate NMDA receptors, when the postsynaptic neuron is depolarized [15], and could thus contribute to NMDA receptor activation-dependent non-Hebbian LTP [2].

### 4.3 Differential expression of synaptic and intrinsic plasticity in the spinal dorsal horn

Numerous reports demonstrate that neurons of the superficial dorsal horn express synaptic plasticity including the presently identified non-Hebbian form of LTP. There are, in contrast, no data to suggest that neurons in the superficial dorsal horn express any form of intrinsic plasticity. Previous studies performed on lamina I or II neurons reported synaptic plasticity [14], [17], [27], but not intrinsic plasticity [3], [11], [41], [54]. In line with this, we also did not find any evidence for intrinsic plasticity in superficial dorsal horn neurons in the present study. Interestingly, in deep dorsal horn neurons, where intrinsic plasticity has been found [24], [33], there are no reports demonstrating synaptic plasticity. Intrinsic plasticity in deep dorsal horn neurons is supposed to affect the transmission of nociceptive information [51], and it has been demonstrated in deep dorsal horn neurons after dorsal root stimulation paired with postsynaptic depolarization [33]. Furthermore, intrinsic plasticity may be induced by spinal nerve ligation in neurons of deep, but not in superficial laminae [49]. This suggests that membrane properties of superficial dorsal horn neurons are apparently more stable than those of deep dorsal horn neurons.

In summary, this is to our knowledge the first study describing a non-Hebbian form of synaptic plasticity in spinal nociceptive pathways. This form of LTP may lead to the generation of heterotopic, secondary hyperalgesia and/or to allodynia and may contribute to opioid-induced hyperalgesia.

## Conflict of interest statement

The authors report no conflict of interest.

## References

[1] Akaike N., Kostyuk P.G., Osipchuk Y.V. (1989). Dihydropyridine-sensitive low-threshold calcium channels in isolated rat hypothalamic neurones. J Physiol.

[2] Alonso A., de Curtis M., Llinás R. (1990). Postsynaptic Hebbian and non-Hebbian long-term potentiation of synaptic efficacy in the entorhinal cortex in slices and in the isolated adult guinea pig brain. Proc Natl Acad Sci U S A.

[3] Balasubramanyan S., Stemkowski P.L., Stebbing M.J., Smith P.A. (2006). Sciatic chronic constriction injury produces cell-type-specific changes in the electrophysiological properties of rat substantia gelatinosa neurons. J Neurophysiol.

[4] Berridge M.J. (2006). Calcium microdomains: organization and function. Cell Calcium.

[5] Bowersox S.S., Gadbois T., Singh T., Pettus M., Wang Y.X., Luther R.R. (1996). Selective N-type neuronal voltage-sensitive calcium channel blocker, SNX-111, produces spinal antinociception in rat models of acute, persistent and neuropathic pain. J Pharmacol Exp Ther.

[6] Calin-Jageman I., Lee A. (2008). Ca_v_1 L-type Ca^2+^ channel signaling complexes in neurons. J Neurochem.

[7] Cervero F., Laird J.M., García-Nicas E. (2003). Secondary hyperalgesia and presynaptic inhibition: an update. Eur J Pain.

[8] Chaplan S.R., Pogrel J.W., Yaksh T.L. (1994). Role of voltage-dependent calcium channel subtypes in experimental tactile allodynia. J Pharmacol Exp Ther.

[9] Chen H.S., Chen J. (2000). Secondary heat, but not mechanical, hyperalgesia induced by subcutaneous injection of bee venom in the conscious rat: effect of systemic MK-801, a non-competitive NMDA receptor antagonist. Eur J Pain.

[10] Chen H.X., Hanse E., Pananceau M., Gustafsson B. (1998). Distinct expressions for synaptic potentiation induced by calcium through voltage-gated calcium and N-methyl-d-aspartate receptor channels in the hippocampal CA1 region. Neuroscience.

[11] Chen Y., Balasubramanyan S., Lai A.Y., Todd K.G., Smith P.A. (2009). Effects of sciatic nerve axotomy on excitatory synaptic transmission in rat substantia gelatinosa. J Neurophysiol.

[12] Daoudal G., Debanne D. (2003). Long-term plasticity of intrinsic excitability: learning rules and mechanisms. Learn Mem.

[13] Devor M., Wall P.D. (1990). Cross-excitation in dorsal root ganglia of nerve-injured and intact rats. J Neurophysiol.

[14] Drdla R., Gassner M., Gingl E., Sandkühler J. (2009). Induction of synaptic long-term potentiation after opioid withdrawal. Science.

[15] Espinosa F., Kavalali E.T. (2009). NMDA receptor activation by spontaneous glutamatergic neurotransmission. J Neurophysiol.

[16] Faber D.S., Korn H. (1991). Applicability of the coefficient of variation method for analyzing synaptic plasticity. Biophys J.

[17] Fenselau H., Heinke B., Sandkühler J. (2011). Heterosynaptic long-term potentiation at GABAergic synapses of spinal lamina I neurons. J Neurosci.

[18] Gold M.S., Gebhart G.F. (2010). Nociceptor sensitization in pain pathogenesis. Nat Med.

[19] Hebb D.O. (1949).

[20] Heinke B., Balzer E., Sandkühler J. (2004). Pre- and postsynaptic contributions of voltage-dependent Ca^2+^ channels to nociceptive transmission in rat spinal lamina I neurons. Eur J Neurosci.

[21] Heinke B., Sandkühler J. (2005). Signal transduction pathways of group I metabotropic glutamate receptor–induced long-term depression at sensory spinal synapses. PAIN®.

[22] Hirasawa M., Pittman Q.J. (2003). Nifedipine facilitates neurotransmitter release independently of calcium channels. Proc Natl Acad Sci U S A.

[23] Hong M., Ross W.N. (2007). Priming of intracellular calcium stores in rat CA1 pyramidal neurons. J Physiol.

[24] Hounsgaard J., Kjaerulff O. (1992). Ca^2+^-mediated plateau potentials in a subpopulation of interneurons in the ventral horn of the turtle spinal cord. Eur J Neurosci.

[25] Huang L.Y.M. (1989). Calcium channels in isolated rat dorsal horn neurones, including labelled spinothalamic and trigeminothalamic cells. J Physiol.

[26] Ikeda H., Heinke B., Ruscheweyh R., Sandkühler J. (2003). Synaptic plasticity in spinal lamina I projection neurons that mediate hyperalgesia. Science.

[27] Ikeda H., Stark J., Fischer H., Wagner M., Drdla R., Jäger T., Sandkühler J. (2006). Synaptic amplifier of inflammatory pain in the spinal dorsal horn. Science.

[28] Inquimbert P., Bartels K., Babaniyi O.B., Barrett L.B., Tegeder I., Scholz J. (2012). Peripheral nerve injury produces a sustained shift in the balance between glutamate release and uptake in the dorsal horn of the spinal cord. PAIN®.

[29] Jhamandas K.H., Marsala M., Ibuki T., Yaksh T.L. (1996). Spinal amino acid release and precipitated withdrawal in rats chronically infused with spinal morphine. J Neurosci.

[30] Jones T.L., Sorkin L.S. (2004). Calcium-permeable a-amino-3-hydroxy-5-methyl-4-isoxazolepropionic acid/kainate receptors mediate development, but not maintenance, of secondary allodynia evoked by first-degree burn in the rat. J Pharmacol Exp Ther.

[31] Kato H.K., Watabe A.M., Manabe T. (2009). Non-Hebbian synaptic plasticity induced by repetitive postsynaptic action potentials. J Neurosci.

[32] Kawamata M., Omote K. (1996). Involvement of increased excitatory amino acids and intracellular Ca^2+^ concentration in the spinal dorsal horn in an animal model of neuropathic pain. PAIN®.

[33] Kim D.K., Kwak J., Kim S.J., Kim J. (2008). Long-lasting enhancement in the intrinsic excitability of deep dorsal horn neurons. PAIN®.

[34] Klein T., Magerl W., Hopf H.C., Sandkühler J., Treede R.D. (2004). Perceptual correlates of nociceptive long-term potentiation and long-term depression in humans. J Neurosci.

[35] Klein T., Magerl W., Treede R.D. (2006). Perceptual correlate of nociceptive long-term potentiation (LTP) in humans shares the time course of early-LTP. J Neurophysiol.

[36] Kohno T., Moore K.A., Baba H., Woolf C.J. (2003). Peripheral nerve injury alters excitatory synaptic transmission in lamina II of the rat dorsal horn. J Physiol.

[37] Kullmann D.M., Perkel D.J., Manabe T., Nicoll R.A. (1992). Ca^2+^ entry via postsynaptic voltage-sensitive Ca^2+^ channels can transiently potentiate excitatory synaptic transmission in the hippocampus. Neuron.

[38] Liu C.N., Michaelis M., Amir R., Devor M. (2000). Spinal nerve injury enhances subthreshold membrane potential oscillations in DRG neurons: relation to neuropathic pain. J Neurophysiol.

[39] Mayer M.L., Westbrook G.L., Guthrie P.B. (1984). Voltage-dependent block by Mg^2+^ of NMDA responses in spinal cord neurones. Nature.

[40] Morisset V., Nagy F. (2000). Plateau potential-dependent windup of the response to primary afferent stimuli in rat dorsal horn neurons. Eur J Neurosci.

[41] Müller F., Heinke B., Sandkühler J. (2003). Reduction of glycine receptor-mediated miniature inhibitory postsynaptic currents in rat spinal lamina I neurons after peripheral inflammation. Neuroscience.

[42] Napier I.A., Mohammadi S.A., Christie M.J. (2012). Glutamate transporter dysfunction associated with nerve injury–induced pain in mice. J Neurophysiol.

[43] Nowak L., Bregestovski P., Ascher P., Herbet A., Prochiantz A. (1984). Magnesium gates glutamate-activated channels in mouse central neurones. Nature.

[44] Nozaki-Taguchi N., Yaksh T.L. (2002). Pharmacology of spinal glutamatergic receptors in post-thermal injury-evoked tactile allodynia and thermal hyperalgesia. Anesthesiology.

[45] Ohsawa M., Kamei J. (1999). Role of intracellular calcium in thermal allodynia and hyperalgesia in diabetic mice. Brain Res.

[46] Petersen K.L., Jones B., Segredo V., Dahl J.B., Rowbotham M.C. (2001). Effect of remifentanil on pain and secondary hyperalgesia associated with the heat-capsaicin sensitization model in healthy volunteers. Anesthesiology.

[47] Pogatzki E.M., Niemeier J.S., Sorkin L.S., Brennan T.J. (2003). Spinal glutamate receptor antagonists differentiate primary and secondary mechanical hyperalgesia caused by incision. PAIN®.

[48] Randic M., Jiang M.C., Cerne R. (1993). Long-term potentiation and long-term depression of primary afferent neurotransmission in the rat spinal cord. J Neurosci.

[49] Ruscheweyh R., Ikeda H., Heinke B., Sandkühler J. (2004). Distinctive membrane and discharge properties of rat spinal lamina I projection neurones in vitro. J Physiol.

[50] Russo R.E., Hounsgaard J. (1994). Short-term plasticity in turtle dorsal horn neurons mediated by L-type Ca^2+^ channels. Neuroscience.

[51] Russo R.E., Nagy F., Hounsgaard J. (1997). Modulation of plateau properties in dorsal horn neurones in a slice preparation of the turtle spinal cord. J Physiol.

[52] Ryu P.D., Randic M. (1990). Low- and high-voltage-activated calcium currents in rat spinal dorsal horn neurons. J Neurophysiol.

[53] Sandkühler J. (2009). Models and mechanisms of hyperalgesia and allodynia. Physiol Rev.

[54] Schoffnegger D., Heinke B., Sommer C., Sandkühler J. (2006). Physiological properties of spinal lamina II GABAergic neurons in mice following peripheral nerve injury. J Physiol.

[55] Strowbridge B.W., Schwartzkroin P.A. (1996). Transient potentiation of spontaneous EPSPs in rat mossy cells induced by depolarization of a single neurone. J Physiol.

[56] Suzuki T., Kodama S., Hoshino C., Izumi T., Miyakawa H. (2008). A plateau potential mediated by the activation of extrasynaptic NMDA receptors in rat hippocampal CA1 pyramidal neurons. Eur J Neurosci.

[57] Tal M., Eliav E. (1996). Abnormal discharge originates at the site of nerve injury in experimental constriction neuropathy (CCI) in the rat. PAIN®.

[58] Tokuyama S., Zhu H., Wakabayashi H., Feng Y.Z., Ho I.K. (1998). The role of glutamate in the locus coeruleus during opioid withdrawal and effects of H-7, a protein kinase inhibitor, on the action of glutamate in rats. J Biomed Sci.

[59] Treede R.D., Magerl W., Sandkühler J., Bromm B., Gebhart G.F. (2000). Nervous system plasticity and chronic pain.

[60] Warncke T., Stubhaug A., Jørum E. (2000). Preinjury treatment with morphine or ketamine inhibits the development of experimentally induced secondary hyperalgesia in man. PAIN®.

[61] Weng H.R., Chen J.H., Cata J.P. (2006). Inhibition of glutamate uptake in the spinal cord induces hyperalgesia and increased responses of spinal dorsal horn neurons to peripheral afferent stimulation. Neuroscience.

[62] Wilder-Smith O.H.G., Arendt-Nielsen L. (2006). Postoperative hyperalgesia: its clinical importance and relevance. Anesthesiology.

[63] Wyllie D.J., Manabe T., Nicoll R.A. (1994). A rise in postsynaptic Ca^2+^ potentiates miniature excitatory postsynaptic currents and AMPA responses in hippocampal neurons. Neuron.

[64] Yang K., Li Y.Q. (2001). Origins of spontaneous and noxious stimuli-evoked miniature EPSCs in substantia gelatinosa. NeuroReport.

[65] Yatani A., Brown A.M. (1985). The calcium channel blocker nitrendipine blocks sodium channels in neonatal rat cardiac myocytes. Circ Res.

[66] Zhang W., Linden D.J. (2003). The other side of the engram: experience-driven changes in neuronal intrinsic excitability. Nat Rev Neurosci.

[67] Zhuo M. (2009). Plasticity of NMDA receptor NR2B subunit in memory and chronic pain. Mol Brain.

